# Equitable Data Sharing in Collaborative Health Research in Sub‐Saharan Africa: A Translational Bioethics Perspective

**DOI:** 10.1002/eahr.60023

**Published:** 2025-05-06

**Authors:** Pamela Andanda, Johannes Machinya, Takudzwa Mutomba

**Affiliations:** ^1^ Professor of law at the University of the Witwatersrand, Johannesburg; ^2^ Lecturer of Health Sociology at the University of the Witwatersrand, Johannesburg; ^3^ Legal practitioner and a doctoral researcher (PhD candidate) at the University of the Witwatersrand, Johannesburg

**Keywords:** data sharing, clinical research, global health, health research, Sub‐Saharan Africa, translational bioethics

## Abstract

Clinical research is essential for establishing the safety, efficacy, and contextualized effectiveness of medical products. Data from multiple sources such as representative target population studies and health and demographic data obtained through health surveillance systems are required for designing clinical research protocols and for recruitment of participants. In this essay, we review barriers from a complex interplay of ethical, legal, and practical challenges in data governance that hamper sharing health data from these sources in Sub‐Saharan Africa. We suggest that a translational bioethics approach offers a valuable framework for addressing these challenges to bridge the gap between theory and practical application of ethical principles in data governance.

Africa's diverse population offers an attractive environment for clinical research, yet fewer clinical studies are conducted in Africa compared to other, higher income regions.^1^ This makes the continent underrepresented in global clinical research, leaving global health entities with insufficient data on the efficacy of their products for patients in African countries.^2^ These concerns highlight the need to conduct clinical studies in representative target populations to establish the safety, efficacy, and contextualized effectiveness of medical products.^3^ Conducting clinical studies in target populations requires access to information and data from multiple sources. For example, health and demographic data, which are obtained through health surveillance systems used for controlling disease outbreaks, play an important role in identifying target populations.^4^ However, sharing health and demographic data for health research raises challenges related to distinguishing primary from secondary use of data, risks of stigmatization, limited local benefits of data sharing, and overall protection of the rights and interests of data subjects.^5^

## BARRIERS TO EQUITABLE SHARING OF HEALTH RESEARCH DATA FROM TARGET POPULATIONS

Equitable data sharing in collaborative health research in Sub‐Saharan Africa (SSA) is essential for advancing health research, particularly clinical studies in target populations that aim to address pressing health challenges such as HIV/AIDS, tuberculosis (TB), malaria, and more recently, Covid‐19. However, health data sharing in SSA—where research often involves collaborations between the Global North and Global South—is fraught with a complex interplay of ethical, legal, and practical challenges, compounded by geographical power imbalances in knowledge production. For instance, historical inequities in global health research often relegate Global South researchers to mere data collectors, while their Global North counterparts dominate data analysis, interpretation, and publication.^6^ This imbalance not only marginalizes African researchers in global health discourse, but it also perpetuates a system where resource‐rich nations disproportionately benefit from research, leaving countries in SSA with limited access to the insights and advancements derived from their own data.^7^

Although progress in data sharing has been reported in SSA, particularly within disease‐specific consortia such as TB, challenges persist in ensuring timely data sharing, not only within the TB community,^8^ but also in studies on malaria and HIV/AIDS. These challenges stem from weak institutional frameworks,^9^ concerns over privacy and confidentiality breaches that could erode trust,^10^ and fears of secondary use of deidentified data, which may contribute to stigmatization of specific communities, populations, or countries.^11^ While some governance structures exist to promote data sharing, sustainable and ethical data sharing practices require the establishment of robust incentive structures that balance research collaboration and compliance with ethical obligations.^12^

One of the most significant barriers to responsible data sharing in collaborative research is the absence of robust, contextually relevant, and cross‐cutting data governance frameworks in SSA. Policies and processes such as “consent, governance processes, data sharing policies and approaches to capacity building” that enable researchers to honor obligations to research participants are needed to ensure ethical data sharing.^13^ While countries such as South Africa and Kenya have enacted comprehensive data protection laws, many others in the region still lack mandatory frameworks for electronic data sharing, leading to inconsistencies in data management practices for data shared across countries.^14^ The absence of clear guidelines often results in researchers sharing data without adhering to established privacy safeguards, increasing the risk of data breaches and unethical use of sensitive health information. Moreover, inconsistencies in the approval processes of data sharing—whether overseen by ethics, scientific, or data sharing committees^15^—can create delays and erode trust among research collaborators.

In addition to governance gaps, resource constraints and systemic underinvestment in research infrastructure further complicate data sharing in SSA. Many researchers in the region lack access to the necessary training, tools, and infrastructure, limiting their ability to manage and share data effectively. For instance, chronic underinvestment in universities and research institutions has resulted in suboptimal training programs, leaving researchers ill‐equipped to navigate the complexities of data‐intensive research.^16^ Moreover, infrastructure challenges, including unreliable power supply and limited internet connectivity, hinder effective data collection, processing, and sharing,^17^ as seen in South Africa, where persistent power outages have disrupted research activities and slowed progress in digital access.^18^ These resource limitations not only impede the production of standardized, high‐quality data but also restrict researchers from sharing data, thereby undermining efforts to adopt open science principles, which rely on effective data sharing.^19^ Anane‐Sarpong

**A translational bioethics approach to data sharing should address power imbalances in global health research by promoting equitable data custodianship.**

and colleagues established that African researchers will continue to face disparities in the governance of data‐sharing unless they receive comparable support to that of their global counterparts.^20^

## NAVIGATING THE SURVEILLANCE‐RESEARCH DIVIDE

Surveillance systems collect personal health data passively for public health monitoring purposes. The integration of surveillance data into research environments presents complex ethical challenges relating to interoperability that require careful consideration at multiple levels. The ethical implications of interoperability primarily revolve around concerns regarding patient data privacy, data governance and security, informed consent, transparency, and equitable access to information, where sharing data across different systems could lead to potential breaches of confidentiality if not properly managed with robust safeguards and clear guidelines for data usage.^21^ These challenges can be particularly acute when data are used for research on vulnerable populations. Repurposing this data for health research thus becomes problematic. For instance, ethical concerns arise when health data originally collected for public health monitoring purposes are later used for research without obtaining explicit consent from the individuals whose information is being used.

Apart from ethical concerns, limited data management skills make it difficult to maintain quality data that are obtained from longitudinal individual‐level surveillance.^22^ Inadequate and low‐quality data in Africa have impeded the understanding of health‐related issues and affected the management and assessment of health care policies, plans, and strategies.^23^ Low‐quality data can widen existing health disparities by limiting equitable access to surveillance benefits and excluding certain populations from health research.

## EQUITABLE DATA SHARING: A TRANSLATIONAL BIOETHICS PERSPECTIVE

A translational bioethics lens offers a valuable framework for addressing the complexities of data sharing, thus bridging the gap between theory and practical application of ethical principles. It ensures that ethical principles—such as privacy, consent, and equity—are not merely theoretical ideals but are meaningfully integrated into real‐world data governance practices, fostering responsible and sustainable data‐sharing frameworks.^24^ This approach is particularly crucial in health research, where high stakes demand the rigorous application of ethical principles. Failure to translate these principles into practice can have serious consequences, including privacy violations, loss of public trust, and potential harm to individuals and communities. By ensuring that ethical considerations are embedded in data governance frameworks, a translational bioethics approach enhances both the integrity and impact of health research. Such frameworks should incorporate clear guidelines on consent protocols, data anonymization, and the ethical use of data, alongside mechanisms for monitoring and enforcing compliance with these standards.^25^

Furthermore, a translational bioethics approach should address power imbalances in global health research by promoting equitable data custodianship. This should involve policy interventions ensuring that African researchers are not merely data collectors, but play a substantive role in data analysis, interpretation, and dissemination of research findings. To achieve this, international funders and collaborators should be required to adhere to principles of reciprocity, ensuring that the benefits of research are equitably distributed between the Global North and the Global South.^26^ Such measures would counteract historical inequities in global health research and create a more collaborative, equitable, and inclusive research ecosystem by translating ethical principles of reciprocity, solidarity, transparency, and accountability into practice.

The principle of reciprocity is significant in the ethical discourse on data sharing in global health research, particularly in addressing persistent power asymmetries between the Global North and Global South. Conditional data‐sharing frameworks that ensure equitable benefits while safeguarding low‐ and middle‐income countries (LMICs) from punitive consequences can help redress these imbalances.^27^ Historically, data‐sharing agreements have favored high‐income countries (HICs), granting them access to, and control over, data collected in the Global South, often without ensuring commensurate benefits for the data‐originating countries in terms of capacity‐building, authorship, or policy influence. This extractive model undermines ethical collaboration and reinforces epistemic injustice, where African researchers remain marginalized in knowledge production. In practical terms, reciprocity requires acknowledging and benefiting data originators and research participants.^28^ Researchers in SSA often prefer data‐sharing arrangements within the region, as this supports local capacity‐building.^29^ Such regional exchanges foster mutual benefit, where data recipients are more likely to reciprocate by sharing their data in return. Conversely, the absence of reciprocal data‐sharing structures in global health research is perceived as exploitative of primary communities and local researchers,^30^ highlighting the need for ethical and equitable governance frameworks.

Solidarity is another fundamental principle in ethical data‐sharing practices, yet its absence in global health data sharing has led to significant inequities. A striking example of the lack of solidarity and reciprocity in data sharing was South Africa's early disclosure of the Omicron Covid‐19 variant. Despite adhering to international norms of scientific cooperation and public health responsibility, South Africa faced punitive travel bans from powerful Global North nations.^31^ Rather than being commended for its transparency, the country was penalized, discouraging future timely data‐sharing efforts by other countries facing similar circumstances. Such incidents expose the geopolitical vulnerabilities in data sharing initiatives when reciprocity is not formally embedded in governance frameworks.

A translational bioethics approach to data sharing requires institutionalizing reciprocity as a foundational principle in global health research. This requires enforceable policies that prevent LMICs from being disadvantaged when they share health data. For instance, global governance frameworks should mandate benefit‐sharing mechanisms,^32^ ensuring that researchers and institutions from LMICs receive tangible advantages when they contribute valuable health data. Moreover, legally binding agreements should be introduced to prevent unilateral punitive actions against countries that share data, fostering a climate of trust, fairness, and ethical collaboration. By embedding reciprocity into data‐sharing policies, a translational bioethics approach would transform current global health research dynamics into a more equitable, transparent, and mutually beneficial system. This shift would not only promote trust among international research partners but also enhance global health security, as countries would be more willing to share crucial health data without fear of economic or political repercussions. Ultimately, responsible data‐sharing practices must move beyond mere ethical rhetoric and toward structural reforms that ensure fairness, equity, and mutual benefit in global health collaborations.

Transparency and accountability are the other fundamental principles in fostering responsible data‐sharing practices in global health research. In many SSA countries, weak regulatory frameworks, ineffective oversight structures, and the absence of clear, standardized policies at both institutional and national levels have created significant barriers to effective data sharing. This has led to a climate of distrust, where researchers prioritize data protection over collaboration due to concerns about governance gaps and potential misuse. Researchers, therefore, hesitate to share data due to legitimate concerns about privacy breaches, intellectual property rights, and the historical misuse of African‐generated data by external entities within the broader research ecosystem.^33^ In such circumstances, a translational bioethics approach offers a practical framework for addressing these challenges by advocating for transparent, accountable, and ethically grounded data‐sharing mechanisms. Trust‐building must be embedded in institutional and national governance structures, ensuring that data‐sharing policies align with international ethical standards. One key solution is the establishment of clear data management plans, which define rules for data access, use, and storage while safeguarding privacy and security. Lack of trust regarding future use of data and potential misuse of shared data, especially when recipients of data lack sufficient understanding of the data or its context, are concerns that need to be addressed particularly where stakeholders fear that lack of proper data governance can lead to an abuse of their trust.^34^ Upholding ethical principles and ensuring integrity in collaborative research are important for building trust among researchers, research participants and their communities.^35^ Most SSA researchers are more inclined to share data on platforms where governance is ensured, and data privacy can be guaranteed.^36^

Accountability mechanisms should also include mandatory reporting structures where researchers are required to document how shared data is being used, by whom, and why. Such measures not only enhance transparency but also safeguard against data misuse. These measures also promote equitable participation in knowledge production by ensuring that data‐sharing practices do not disproportionately favor well‐resourced institutions in the Global North at the expense of local researchers and communities. Enforcing these principles through legally binding agreements and institutional review processes would empower African researchers, enabling them to engage in open science while safeguarding their intellectual contributions and the rights of research participants.

In sum, applying a translational bioethics lens to data governance in SSA would help bridge the gap between ethical theory and practice, ensuring that data‐sharing policies are both practically viable and ethically sound, fostering a culture of equitable collaboration, fairness, and trust in global health research. It is also important to mobilize resources to establish governance frameworks and develop guidelines that enable equitable data sharing. Capacity building is essential in areas such as data governance, data privacy regulation, and data quality assurance to enable African researchers to share data in a timely way.

## ACKNOWLEDGMENTS

This work was supported, in whole, by the Gates Foundation (Grant number INV‐058417). The conclusions and opinions expressed in this work are those of the authors alone and shall not be attributed to the Foundation. We gratefully acknowledge research assistance from Advocate Larisha Bedhesi, a PhD candidate at the University of the Witwatersrand, Johannesburg.
